# A positive experience in applying the biolistic approach to potato varieties Aksor and Nevskiy

**DOI:** 10.18699/VJ21.019

**Published:** 2021-03

**Authors:** N.P. Malakhova, Y.A. Skiba, G.A. Iskakova, D.A. Naizabayeva, B.K. Tezekbaeva, G.A. Ismagulova, E.R. Maltseva

**Affiliations:** M.A. Aitkhozhin Institute of Molecular Biology and Biochemistry, Almaty, Kazakhstan Institute of Plant Biology and Biotechnology, Almaty, Kazakhstan; M.A. Aitkhozhin Institute of Molecular Biology and Biochemistry, Almaty, Kazakhstan Institute of Plant Biology and Biotechnology, Almaty, Kazakhstan Almaty Branch of National Center for Biotechnology in the Central Reference Laboratory, Almaty, Kazakhstan; M.A. Aitkhozhin Institute of Molecular Biology and Biochemistry, Almaty, Kazakhstan Institute of Plant Biology and Biotechnology, Almaty, Kazakhstan; M.A. Aitkhozhin Institute of Molecular Biology and Biochemistry, Almaty, Kazakhstan Almaty Branch of National Center for Biotechnology in the Central Reference Laboratory, Almaty, Kazakhstan; M.A. Aitkhozhin Institute of Molecular Biology and Biochemistry, Almaty, Kazakhstan Institute of Plant Biology and Biotechnology, Almaty, Kazakhstan; M.A. Aitkhozhin Institute of Molecular Biology and Biochemistry, Almaty, Kazakhstan Institute of Plant Biology and Biotechnology, Almaty, Kazakhstan Almaty Branch of National Center for Biotechnology in the Central Reference Laboratory, Almaty, Kazakhstan; M.A. Aitkhozhin Institute of Molecular Biology and Biochemistry, Almaty, Kazakhstan Institute of Plant Biology and Biotechnology, Almaty, Kazakhstan Almaty Branch of National Center for Biotechnology in the Central Reference Laboratory, Almaty, Kazakhstan

**Keywords:** biolistic transformation, potato, explant, internodes, calli, biotechnology, биобаллистическая трансформация, картофель, эксплант, междоузлия, каллусы, биотехнология

## Abstract

The method of biological ballistics (biolistic transformation, genetic bombardment) of plants is one of the most modern methods used for direct gene transfer into plant cells. The main advantages of this method include the ability to simultaneously incorporate several target genes into the plant genome, carry out transfer without unnecessary agrobacterial parts and plasmid DNA sequences, and the short time needed to produce transgenic cells. For different plant objects, the efficiency of obtaining transgenic plants by the ballistic method varies from 1 to 3 %. For potato plants, the transformation efficiency is quite low at the moment and the selection of optimal conditions for biolistics is one of the pressing issues of practical biotechnology. This article presents a successful experience of introducing two genes of interest into two potato varieties using the biolistic approach. The results of biolistic transformation experiments are presented for two types of explants: potato internodes and calli of the varieties Aksor and Nevskiy. Of the 862 explants used for transformation, 56 regenerated plants were obtained. PCR screening of transformants revealed one plant with the insertion of the chitinase gene, one with the insertion of the endo-β-1,3-glucanase gene, and co-transformation by both genes was confirmed in four regenerants. The average transformation efficiency for potato explants was 0.7 %. A high number of regenerants (56) as opposed to a low number of transformants (6) reflects an attempt to increase the number of regenerants by using a lower concentration of the selective agent (kanamycin). Although this approach requires more effort, it can be used to produce potato lines with integrated genes of interest for further use in crop breeding. The lines of potato obtained in the current study by introducing two genes associated with the plant response to fungal pathogens will be further assessed for their resistance to fungal diseases and, if successful, will be used in potato crop breeding.

## Abstract (ru)

Метод биобаллистической трансформации растений – один из наиболее современных способов, используемых для прямого переноса генов в клетки растений. К главным преимуществам этого метода относятся возможность одновременно встраивать в геном растений несколько целевых генов, проводить перенос без лишних агробактериальных участков и последовательностей плазмидной ДНК, а также короткие сроки получения трансгенных клеток. В среднем для разных объектов эффективность получения трансгенных растений баллистическим методом варьирует от 1 до 3 %. Для растений картофеля на сегодняшний момент эффективность трансформации довольно низкая, и подбор оптимальных условий проведения биобаллистики является одним из актуальных вопросов практической биотехнологии. В данной статье представлен положительный опыт внедрения двух генов интереса в два сорта картофеля с использованием подходов биобаллистики. Представлены результаты экспериментов по биобаллистической трансформации двух видов эксплантов – междоузлий и каллусов картофеля сортов Аксор и Невский. Из 862 эксплантов, подвергнутых трансформации, получено 56 растений-регенерантов. В результате скрининга трансформантов методом ПЦР выявлено одно растение со вставкой гена хитиназы, одно со вставкой гена эндо-β-1,3- глюканазы и в четырех регенерантах подтверждена котрансформация обоими генами. Средняя эффективность трансформации эксплантов картофеля составила 0.7 %. Большое число растений-регенерантов (56) по отношению к числу трансформантов (6) отражает попытку увеличить количество регенерантов использованием низких концентраций селективного агента (антибиотика канамицина). Хотя этот подход требует заметных усилий, он может применяться для получения линий картофеля со вставкой генов интереса для их дальнейшего использования в селекции. Линии картофеля, полученные в данном исследовании после внедрения двух генов, связанных с устойчивостью растений к грибным патогенам, будут в дальнейшем оцениваться на устойчивость к грибным заболеваниям и в случае успешного прохождения испытаний будут включены в селекционный отбор.

## Introduction

Modern biotechnological studies dedicated to the transformation of potatoes use a number of approaches based on both direct gene transfer and agrobacteria. The agrobacterial approach has traditionally been used to transform potato leaves, internodes, and tubers. This is due to the good susceptibility of potato plants to agrobacteria, and this method serves best when it is necessary to transfer one gene to the genome of the host plant. If the goal of research is the introduction of several genes into the genome at once, with their coordinated integration and expression, then the method of choice will be the biolistic transformation (Romano et al., 2001; Craig et al., 2005).

It is known that the effectiveness of biolistic transformation depends on a large number of factors, including the number of embryogenic cells, their regenerative ability, the number of DNA-coated particles, and the amount of DNA that is bound to the particles (Rivera et al., 2012). However, in case of potatoes the transformation success also largely depends on the genotype of the plant (Jo et al., 2014).

Despite the fact that the method of biolistic transformation has been used for about 20 years and has become routine for some crops (Taylor, Fauquet, 2002), it is still rarely used for potatoes. The first work on the bombardment of potatoes was published in 2001 (Romano et al., 2001), but since then the number of such works has not exceeded a dozen. The sizes of introduced sequences (Ercolano et al., 2004), the comparison with PEG-mediated transformation of protoplasts (Craig et al., 2005) and even the possibility of conducting bombardment with agrobacterial cells carrying three genes of interest were studied (Nguyen et al., 2001).

According to available literature, the efficiency of potato transformation largely depends on the explants for bombardment: for leaves, it is 0.02 plants per transformed explant; this index is higher for micro-tuber slices (0.1 plants per transformed explant), and the maximum result is achieved when using internodes (0.77 plants per transformed explant) (Romano et al., 2001).

However, to date, there are not enough comparable data in the literature on the effectiveness of transformation using the bombardment method depending on the type of explants. The explant type mostly used in the studies where transformation efficiency was indicated in numbers was potato leaves. For example, Craig et al. (2005) indicate 0.5 transformation events per leaf as efficiency, and Nguyen et al. (2001) confirm these numbers, indicating that the transformation efficiency was similar to Craig’s data. The only comparable data available in the literature are the data by Romano et al. (2001)

In addition, all works that somehow describe the effectiveness of transformation state that it depends on the genotype, with a special mention of “low frequency of the appearance of regenerants selected using kanamycin” (Ercolano et al., 2004). The experiments performed by Joe and colleagues (Jo et al., 2014) even show that marker-free transformation is more prone to the characteristics of the variety compared to marker-mediated transformation. The authors suggest that this may be due to the variety-specific features of antibiotic tolerance, which gives transformed cells different opportunities for shoot development.

Although the interest in biolistic transformation for its use in potato breeding increases, more studies on the application of method are needed to establish the most suitable explant type and fine-tune the procedures for both bombardment and post-bombardment stages.

The purpose of this study was to evaluate the effectiveness of the biolistic transformation of potatoes depending on the plant explant type.

## Materials and methods

The object of study was potato of two varieties – Aksor and Nevskiy. Aksor is a potato variety of Kazakhstan’s breed, characterized by relative heat resistance, drought resistance, and disease resistance (http://www.kartofel.org/cultivars/reg_cult/ aksor.pdf). Nevskiy is a potato variety of Russian selection, medium early, high-yielding, poorly resistant to fusarium infection (http://www.kartofel.org/katalog/katalog1.pdf).

**Preparing plant explants for transformation.** Explant type 1. The internodes of test plants (21–28 days old), grown on the medium for test tube plants, were longitudinally cut into segments and directly exposed to OSS osmotic medium with cut side up (composition of the medium is provided below in the corresponding section) 24 hours before the bombardment (Sanford et al., 1993).

Explant type 2. A two-week callus was obtained from potato plants cultivated on the medium for test tube plants, cut along internodes and put with cut side up on callus-inducing medium. Resulting callus was transferred to osmotic medium and simultaneously embryogenic callus was selected with the control of a stereoscopic microscope (Sambrook et al., 1989).

**Genetic constructs for potato transformation.** The fullsize genes – class I chitinase and potato endo-β-1,3-glucanase – were cloned from the potato variety Aksor induced by Fusarium solani (Chirkin et al., 2016). The genes were excised from pUC57 vector by BamHI and SacI sites and independently cloned to pBI121 vector containing the selective kanamycin resistance gene, resulting in two separate genetic constructs. Minimal expression unit (MEU) was excised from the vector with EcoRI and BglII restriction endonucleases.

**Biolistic transformation of potato embryogenic callus and internodes.** The biolistic transformation of the potatoes was carried out on PDS-1000/He Particle Delivery System (Bio-Rad) device at vacuum pressure of 91.4–94.8 kPa, using gold particles bound to DNA according to the manufacturer’s instructions. For each experiment, 100 ng of elution-purified MEU was used. Microparticles (1 μm) of gold (Bio-Rad) coated with DNA according to the binding procedure (Sanford et al., 1993) were used. Four to seven shots were fired for each design, using both 900 and 1100 psi discs

**Nutrient media.** The composition of the nutrient media used in the study is provided in Table 1. All the media are based on Murashige–Skoog medium (Murashige, Skoog, 1962).

**Table 1.**
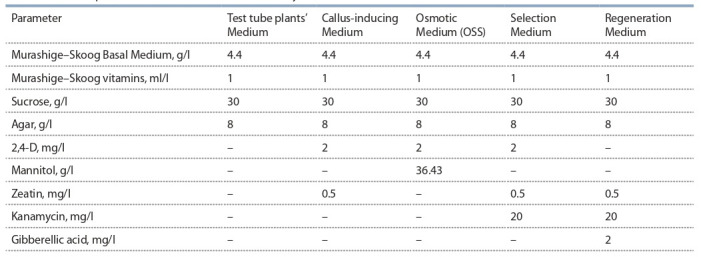
The composition of the media used in the study

**Cultivation of plant tissue after transformation.** After the bombardment, the plant material was left on osmotic media for two days, then the transformed calli were planted on Selection Medium containing antibiotic kanamycin as a selective agent. Selection took from 45 to 65 days with exposition to the light to register points of growth, detected by the presence of chlorophyll-bearing cells at 10–20× magnification. The selected growth points were passaged to regeneration medium. Regenerants were first planted in Petri dishes, and then, as they grew, into Magenta boxes. Resulting individual plants were passaged to test tubes plants’ medium.

**Analysis of the presence of the target gene insert in regenerated plants.** All regenerated plants were analyzed for the presence of the target gene insert. The analysis of the insert was carried out by a polymerase chain reaction using primers selected for the introduced genetic design. For analysis of the presence of the glucanase gene insert in potato plants, the following primers were used: Ch_S_F (CCACG TCTTCAAAGCAAGTGG); Gl_S_R (TGAATGTTGGTG GCAACAAGTAAT).

The primer design for the chitinase gene insert was the following: Ch_S_F (CCACGTCTTCAAAGCAAGTGG); Ch_S_R (CATTTGTATTACCACACCAGCCG).

DNA for analysis was isolated using Sigma EXTRACT-NAMP-RED PLANT PCR kit. Two microliters of the resulting DNA were used for PCR along with 2 ul HotTaq ×10 buffer, 2 mM MgCl2, 0.2 mM dNTP, 10 pmol of each primer and 1 unit of HotTaq DNA polymerase. The regime for the amplification of both genes was the following: initial denaturation at 95 °С for 5 minutes, followed by 35 cycles (94 °С – 30 sec; 55 °С – 30 sec; 72 °С – 40 sec) and 72 °С – 5 minutes.

Visualization of the PCR products was carried out in 1.5 % ethidium bromide-stained agarose gel in TAE buffer and processed by gel documentation system (Bio-Rad).

## Results

**Fig. 1.**
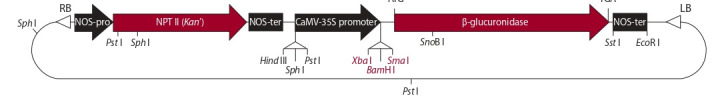
Scheme of pBI121 vector.

For the biolistic transformation, we used two potato varieties (Aksor and Nevskiy) introduced in Kazakhstan and the genetic constructs based on the pBI121 vector (Fig. 1) containing selective kanamycin resistance gene and two target genes – classI chitinase gene and potato endo-β-1,3-glucanase gene – cloned from the potato variety Aksor induced by Fusarium solani infection (Chirkin et al., 2016). These genes were part of the previous research of plants resistance to fungal diseases, with the idea that their constant expression might increase plant resistance to fungal diseases.

The genes were introduced into the vector instead of the second selective marker, the β-glucuronidase gene, under the control of CaMV-35S promoter to ensure constant expression of the genes, allowing the study of their importance in the plant’s immune response and the development of potato plants with enhanced resistance to fungal diseases. An independent construct was created for each gene; the presence of the selective gene in both constructs allowed selecting plants with both genes at once, as well as with individually introduced genes.

To transform potato cells with two target genes at once, we performed a biolistic transformation, in which the co-transformation efficiency is quite high and, according to the available literature (Romano et al., 2001), is up to 85 % when using two constructs simultaneously.

The regenerative ability of potatoes is lost quite quickly during callus formation and cultivation, so, when choosing the optimal tissue for biolistic experiments, we were primarily guided by the presence of the regenerative ability of the callus cultures for a long period necessary for the biolistics itself and subsequent regeneration and selection on a nutrient medium. 

A few studies dedicated to potato biolistic transformation use leaves of 4–5-weekold seedlings as explant material. However, our preliminary experiments have shown insufficient regeneration efficiency of this type of explant on the Aksor and Nevskiy varieties. It should be noted that explants obtained from internodes have high regeneration potential, and therefore have been successfully used in a number of studies on agrobacterial transformation to improve the efficiency of the process (Kisgyörgy et al., 2008; Mielby et al., 2012).

Our preliminary biolistic experiments showed higher effectiveness of internodes as explant in comparison with leaves and apical meristems. In this regard, to study the effectiveness of the potato biolistic transformation depending on the type of plant explant, we selected two types of explant – internodes and 2–3-week calli obtained from potato internodes

**Fig. 2.**
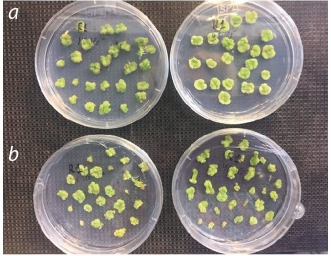
Embryogenic callus of potato on a nutrient medium for the callus induction: a – calli of Aksor potato; b – calli of Nevskiy potato.

It should be noted that the two potato varieties selected as the starting material – Aksor and Nevskiy – differed in behavior in cell culture. During the cultivation of internodes on a callus-inducing nutrient medium (Fig. 2), the embryogenic calli of Aksor potatoes were large, uniform, while the calli of Nevskiy potato did not reach large sizes and began to turn yellow and die relatively quickly

**Fig. 3.**
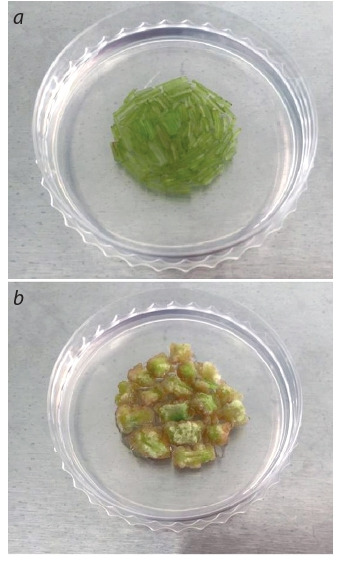
Potato explant on an osmotic biolistic medium: a – fresh

Both types of explants – fresh potato internodes and a 2–3-weeks-old callus obtained from potato internodes (Fig. 3) – were used for the biolistic experiment

This approach was supposed to offset the difference in callus formation of explants taken in the experiment. This was especially important for calli of the Nevskiy variety as they showed insufficient callus formation from the original plant material, according to the results of the visual assessment of their ability to start callus formation in vitro.

A total of 5 series of transformation experiments was conducted, each containing 10 plates, with a total number of explants used – 862, of which 475 explants were obtained from the Aksor variety, and the remaining 387 – from the Nevskiy variety. In the presence of the selective agent, cells carrying the antibiotic resistance gene survived, while non-transformed cells died. Kanamycin, an antibiotic widely used in the potato transformation studies, was used as a selective agent at a concentration of 20 mg/l. By the end of the selection stage, during which callus was induced in the fresh internodes used for ballistics, the two types of explants were almost equal in shape and size.

**Fig. 4.**
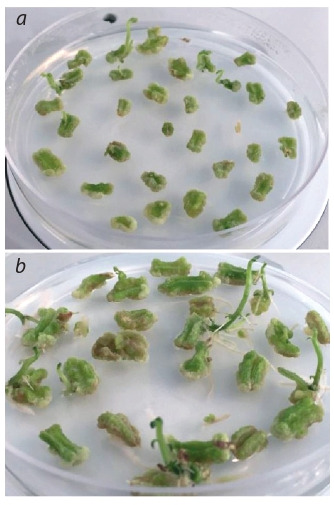
Potato explants after biolistic transformation: a – рotato explants after exposure to selective medium; b – рotato explants at the regeneration stage.

After subculturing the explants on the selection medium for 2 months, the plates were visually analyzed for the presence of the developing plantlets (points of growth), which were transferred to regeneration medium. Only calli with visible signs of regeneration were selected, as in Figure 4, a, which shows the explants to be transferred to regeneration medium (see Fig. 4, b) to stimulate the regenerative abilities of the material by zeatin and gibberellic acid. The regenerant plants were grown and subcultured on test tube plants’ medium for further study and propagation

**Fig. 5.**
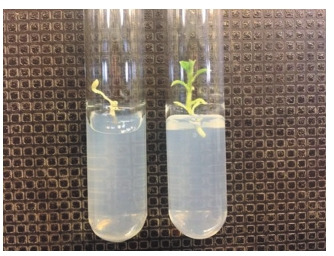
Regenerant plants on selective medium in the second stage of selection.

In general, the explants’ regenerative ability was sufficient, but not all visible seedlings reached the stage of separation from the main callus into separate magenta boxes or subsequent transfer to separate tubes. After separation from the main callus, some regenerants acted differently in the second round of selection. As shown in Figure 5, one of the plants (right) is tolerant to the presence of kanamycin, while the other (left) did not go through the selection stage and was rejected.

As a result of the selection, potato regenerant plants presumably carrying an insert of target chitinase and glucanase genes were obtained. The number of potato regenerant plants obtained after the transformation of various types of explants (internode and callus) was different for the two varieties. Atotal of 56 regenerants was obtained from the 862 explants taken in the experiment, with the largest number of regenerants from the internodes of the Nevskiy variety. The survival of regenerated plants from the Aksor callus tissue was 7.4, and that of the Nevskiy variety was 4.8 (Table 2).

**Table 2.**
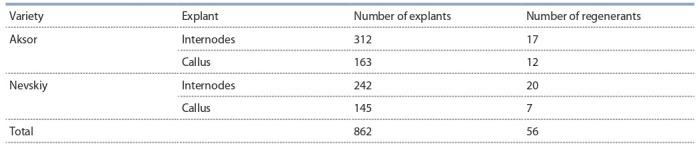
The number of potato regenerants on Selection Medium

The screening of plants was performed after co-transformation. The resulting regenerated potato plants of both varieties were propagated microclonally in vitro. For each of the 56 transformed plants (lines), 10 test tube plants were obtained, which were used to perform polymerase chain reaction (PCR) for the presence of the target gene insert. Part of the leaf was cut from the plants for DNA isolation, and subsequently used for PCR with insert-specific primers.

The analysis of potato regenerants was carried out by PCR with specific primers. Since the introduced genes were also originally isolated from potatoes, the primers were designed in a special way: the forward sequence of the primer was annealing to the promoter part of the construct, and the reverse – with chitinase and glucanase genes, respectively, leading to two reaction products of 290 bp (chitinase gene) and 210 bp (glucanase gene).

**Fig. 6.**
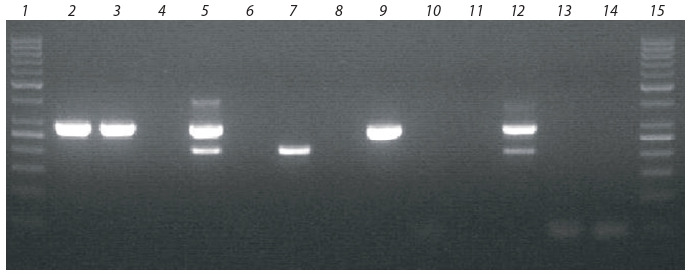
Electrophoretic analysis of potato regenerated plants for the presence of chitinase and glucanase gene inserts. Lane 1 and 15 – molecular weight marker (50 bp), lane 12 – positive control, lanes 13, 14 – negative controls, lanes 2–11 – test samples: 2, 3 and 9 – Aksor line carrying chitinase gene (DNA extracted from three plants of one line), 5 – Nevskiy line with chitinase and glucanase genes, 7 – Nevskiy line with glucanase insert, 4, 6, 8 – Aksor lines with no inserts, 10, 11 – Nevskiy lines with no inserts.

Of the 56 potato regenerant plants, one plant carried an insert of the chitinase gene (Aksor variety, explant type – fresh internodes), one carried an insert of the glucanase gene (Nevskiy variety, fresh internodes), and four more were characterized by the presence of two genes (two belonged to Aksor variety, fresh internodes, one to Aksor variety, callus, and one to Nevskiy variety, callus). An example of the PCR confirmation results of the target genes’ insertion is given in Figure 6.

Thus, the data of PCR analysis show that out of 862 explants of potato internodes and calli, only six plants were successfully transformed as the result of biolistic transformation; these plants carry either one or both inserts of the target chitinase and glucanase genes. Transformation efficiency was assessed by the number of viable regenerated plants relative to the number of explants subjected to biolistics (Table 3). 

**Table 3.**
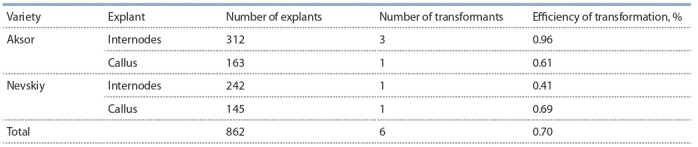
The transformation efficiency of potato varieties Aksor and Nevskiy

As seen from the data presented in Table 3, the average transformation efficiency for both varieties of potatoes was 0.7 %. At the same time, the maximum transformation efficiency was achieved using fresh internodes of the Aksor variety (0.96 %), and the minimum – with fresh internodes of the Nevskiy variety. It should be noted that in experiments with Aksor potatoes, the minimum level of biolistics efficiency on calli taken as explants (0.61 %) slightly differs from the maximum value of transformation efficiency for the Nevskiy variety on internodes – 0.41 %, which indicates the difference in characteristics for each varieties of potatoes.

In experiments on the co-transformation of potatoes 4 out of 6 regenerant plants were co-transformed, which is 66.7 %. These results confirm the data of other authors on the high degree of joint integration of two separate genes of interest (Romano et al., 2001).

## Discussion

This study was aimed to compare the effectiveness of the biolistic transformation using different explant types and depending on the potato genotype. At the same time, the low number of transformed plants does not let us draw solid conclusions. It is doubtless that for the biolistic transformation it is necessary to consider the individual characteristics of the potato variety and experimentally select a specific explant type. Moreover, even with a low number of transformants we can state that fresh internodes are best suited for transforming the Aksor variety, while embryogenic callus should be selected for the Nevskiy variety. This finding will be used in future works dedicated to biolistic transformation for potato crop breeding

The average transformation efficiency was not very high (0.7 %), which corresponds with the findings of other authors. At the same time, it seems that some transformed embryogenic callus might be lost at the selection and regeneration stages, so adaptation of these procedures will help raise the efficiency index. One of the attempts to do so was done in the current work – we ref lected on the concern of other authors that selection with kanamycin adversely inf luences the regenerants’ appearance and took the lower limit of the selective agent’s concentration – kanamycin was used at 20 mg/l, whereas in some sources up to 100 mg/l is recommended. This explains the high number of regenerants as opposed to the number of transformants. The decision on kanamycin’s concentration was taken after the preliminary experiments on stable transformation (data not shown), in which no regenerants were obtained after the selection stage, while the experiments on transient transformation showed sufficient number of transformed cells (data not shown). Thus, further research is needed for the post-bombardment stage to make the transformation event more successful. Although kanamycin is one of the most used antibiotics for potato transformation, other selective agents should be explored, including such options as herbicide resistance agents, which are also a valuable trait for crop breeding and will help in agricultural crop production.

The high co-transformation efficiency justifies the use of this method for introduction of several genes of interest in one shot. Further studies should be dedicated to introducing more than two genes at once for the evaluation of this approach’s efficiency. Combined, the protocol of adapted selective/regeneration stage and biolistic technique with more than two genes of interest at once will provide potato crop breeding with valuable efficient and time-saving methods.

## Conclusion

Despite the fact that agrobacterial transformation remains the most used method of introducing new genes into potato, the biolistic transformation method has certain advantages depending on the goals of the transformation. For example, with the help of bombardment, several genes can be introduced at once, and this can be done both as a single cassette and as co-transformation, the efficiency of which is quite high. This study showed high co-transformation efficiency of two independent genetic constructs, and will serve as a basis for further studies dedicated to introduction of more independent genetic constructs at once. Considering the insufficient number of published works on stable biolistic transformation of potatoes, we believe that data obtained in this study will make a certain contribution to the development of biolistic transformation technology of potatoes.

At the same time, the lines produced in the course of this work will be used to evaluate their resistance to fungal diseases, and, if successful, can be used in potato crop breeding for increased resistance to fungal diseases.

## Conflict of interest

The authors declare no conflict of interest.
